# A Novel Neutrosophic Method for Automatic Seed Point Selection in Thyroid Nodule Images

**DOI:** 10.1155/2019/7632308

**Published:** 2019-04-10

**Authors:** S. O. Haji, R. Z. Yousif

**Affiliations:** Department of Physics, College of Science, Salahaddin University, Hawler, Iraq

## Abstract

The thyroid nodule is one of the endocrine issues caused by an irregular cell development. This rate of survival can be improved by earlier nodule detection. Accordingly, the accurate recognition of the nodule is of the utmost importance in providing powerful results in building the survival rate. The reduction in the accuracy of manual or semiautomatic segmentation methods for thyroid nodule detection is due to many factors, basically, the lack of experience of the sonographer and latency of operation. Most lesion regions in ultrasound images are homogeneous. Therefore, the value of entropy in these regions is high compared to its neighbours. Based on this criterion, a novel procedure for automatically selecting the seed point in thyroid nodule images is proposed. The proposed system consists of three components: neutrosophic image enhancement and speckle reduction to reduce speckle noise and automatic seed selection algorithm extracted from the centre of candidate block in ultrasound thyroid images based on the principle that most of its Higher Order Spectra Entropies (HOSE) from Radon Transform (RT) at different angles are within the range between average and maximum entropies, and the region growing image segmentation is applied with the constant threshold. The performance of proposed automatic segmentation method is compared with other methods in terms of calculating, True Positive (TP) value (96.44 ± 3.01%), False Positive (FP) value (3.55 ± 1.45%), Dice Coefficient (DC) value (92.24 ± 6.47%), Similarity Index (SI) (80.57 ± 1.06%), and Hausdroff Distance (HD) (0.42 ± 0.24 pixels). The proposed system can be considered as an added value to the malignancy diagnosis in thyroid nodule by an endocrinologist.

## 1. Introduction

Thyroid nodule malignancy is one of the vital life-threatening issues that occurred due to irregular growth of cells that might be benign or malignant [1]. As categorized by American Cancer Society's evaluations for thyroid malignancy in 2018, out of 53,990 new instances of thyroid cancer, 13,090 were males and 40,900 were females in the United States [2].

For an endocrinologist, the basic problem is to physically identify the exact thyroid nodule in the ultrasound image and classify it as benign or malignant [3]. Computer-aided detection frameworks are becoming increasingly popular and help endocrinologists make accurate decisions to understand an enormous amount of image information [4]. One of the main difficulties to be considered in designing a fully computerized recognition framework is the accurate representation of nodules with automatic extraction of the region of interest (ROI) within the thyroid organ. Alternative difficulties are speckle noise suppression in ultrasound images which was addressed in this study.

Koundal et al. [5] in 2018 utilized full automated computer-aided detection framework for speckle reduction and segmentation of nodules from thyroid ultrasound images. Speckle is an unfortunate obstruction impact, happening when at least two ultrasound waves interfere with each other, constructively or destructively, producing bright and dark spots [6]. For preprocessing of ROI speckle reducing anisotropic diffusion (SRAD) filter has been used by Yu et al. [7]. SRAD is a filter, which iteratively reduces speckle noise, preserves edges, and simultaneously enhances the contrast of the image.

In recent decades, further studies have been carried out to remove speckle noise, e.g., the nonlocal means (NLM) filter utilized by Avazpour 2009 [8] and anisotropic diffusion (SRAD) filter proposed and created by Mat Isa et al., 2006 [9].

Recently, numerous researchers have used neutrosophy in various applications such as image noise reduction and segmentation, which have shown that the theory of neutrosophy provides a good execution due to its indeterminacy and performance [10–13].

The proposed technique of thyroid nodule summation is mainly based on entropies derived after the application of radon transformation using HOS. Its spectra sometimes referred to as poly-spectra are spectral representations of higher order statistics, i.e., moments, and cumulated by third order and beyond. HOS was first applied to real signal processing problems in 1970. It may be more advantageous to analyze biomedical signals because it is nonlinear, nonstationary, and non-Gaussian [14]. HOS has been applied to various applications, for instance, 1D pattern recognition [15], array signal processing [16], and ultrasound image processing [17].

A seed is a test pixel with a perfect trademark that belongs to the suspicious region and ought to be the piece of the region of interest [18]. Since the region growing outcome is delicate to the underlying seeds, the precise seed choice is essential for image segmentation [19].

Segmentation is a standout of the most troublesome and essential assignments in medical image processing. This dynamic field of research throughout the most recent two decades makes a basic organization or format of the medical image, to indicate a region of interest (ROI). Segmentation is the way toward apportioning image into a few areas as per particular standards. The reason for segmentation is utilizing these regions for ROI detection to recognize any irregularities or lesions. Nature of segmentation decides the possible achievement or disappointment of the investigation or analysis.

Segmentation of medical images utilizing seeded region growing (SRG) procedure is progressively turning into a well-known technique as a result of its capacity to include abnormal state information of anatomical structures in seed choice process [20]. Poonguzhali and Ravindran [21] depict a fully automatic technique for segmentation of masses on ultrasound images by utilizing region growing technique. In their work, the region growing begins by selecting a seed pixel, followed by adding new pixels to the segment (augmented fragment) until the segmentation standard is fulfilled [22]. Seeded region growing has the benefit of indicating only one interested region by putting a seed in it. Be that as it may, the execution of SRG calculations relies on this position.

Various works can be discovered with respect to automatic seed selection. Michahial et al. [23] proposed a technique to calculate and recognize the seed point automatically by which automatic contour initialization is done. Chang et al. in 1994 [24] and Avazpour et al. in 2009 [8] pointed out how the Histogram Feature technique can be used in seeded selection based on feature extraction approach. Mat Isa et al. in 2006 [9], Saad et al. in 2012 [25], and Al-Faris et al. in 2014 [26] utilized moving K-means technique for seeding selection based on region extraction, respectively. On the other hand, Mustafa et al. in 2010 [27] used active contour model for seed selection based on edge extraction approach.

The idea presented in this work is to identify a seed pixel from the abnormal regions based on entropy capability in differentiating between nonhomogeneous and homogeneous regions in ultrasound images. The previous techniques were depending on entropies derived from spatial domain [21]. HOS is employed as a nonlinear method that helps to capture the subtle changes in pixels of the image and hence to identify the seed point. It was noticed that HOS methods would be a superior methodology than the conventional time domain and frequency domain methods in analyzing the biosignals. It is a great apparatus for the nondirect dynamical investigation of the biomedical signals, so as the case it can battle commotion and give great outcomes even with the case it can combat noise and give good results even with weak signals. In another place, HOS is valuable in recognizing nonlinear coupling and deviation from Gaussianity and features obtained from it tend to be made invariant to move, rotation, and enhancement. The new approach in this research is to depend on the important entropies' values, namely, phase entropy and bispectrum entropies using HOS, these parameters can be used to select a suitable seed pixel from the suspicious regions. The proposed system is beginning with applying Radon transform to each image block; then HOS based entropies values extracted from different ultrasound image resulting from blocks at different orientations (angles) were carried out. Finally, we select the center of candidate block for which most of its entropy's features are high (above the average value derived from all blocks). The spectral entropies are high for a homogeneous region of the possible lesion and low for nonhomogeneous regions. These feature scans are explored for numerous healthcare applications.

## 2. Materials and Methods

### 2.1. Dataset

The ultrasound image dataset has been utilized in this paper to compute the efficiency of proposed segmentation method from the open access Digital Database of thyroid ultrasound images from the Universidad Nacional de Colombia Laboratory [28]. It consists of 92 thyroid ultrasound images, out of which 50 were males and 42 were females with various ages. The images were extracted from thyroid ultrasound video sequences captured with a TOSHIBA linear transducer. Thyroid nodules images are saved in ultrasonography system that includes a complete annotation and diagnostic description of suspicious thyroid lesions, using the TI-RADS lexicon description performed by at least two expert radiologists.

### 2.2. Pre-Processing

#### 2.2.1. Image Enhancement and Sharpening

The principal goal of an image enhancement is to draw out the hidden image details or to expand the image contrast from another powerful range [29]. Neutrosophic based image enhancement is used in this research. Neutrosophy is a part of philosophy displayed in [30] as a generalization of dialectics and studies the origin, nature, and extent of neutralities, close to their interactions with different ideational spectra. In neutrosophy hypothesis, only one out of every event has a specific level of reality, in addition to a misrepresentation degree and an indeterminacy degree that must be considered uninhibitedly from each other.

*(1) The Image in Neutrosophic Set*. Assume U is a universe of discourse and W is an arrangement of U, which is made by bright pixels. A neutrosophic image PNS is described by three participation sets* T, I, F*. A pixel P in the image is described as P(*T*,* I*,* F*) and related to W in an accompanying way: It is t, true, in the set, i, indeterminate in the set, and f, false, in the set, where t varies in* T*, i varies in *I*, and f varies in *F*. The component pixels' functions* TC*,* IC,* and* FC* are calculated to transform image from gray-level domain to neutrosophic domain. The participant functions as mentioned before are computed [31], as shown below:

(1) $$\begin{array}{c} TC=\frac{{\overset{⌢}{q}}_{ij}-{\overset{⌢}{q}}_{min}}{{\overset{⌢}{q}}_{max}} \end{array}$$

where i varies from 0 to n-1, j varies from 0 to m-1, ${\overset{⌢}{q}}_{ij}$ is the local mean obtained using a window, ${\overset{⌢}{q}}_{min}$ is the minimum grey level value, and ${\overset{⌢}{q}}_{max}$ is the maximum grey level value.

(2) $$\begin{array}{c} {\overset{⌢}{q}}_{ij}=\frac{1}{w\times w}\overset{i+w/2}{\underset{m=i-w/2}{\sum }}\overset{j+w/2}{\underset{n=j-w/2}{\sum }}q_{mn} \end{array}$$

where *q*_*mn*_ is the noisy image and *w* is the size of the window.

(3) IC=δij−δminδmax

(4) δij=absqij−q⌢ij

where *δ*_*ij*_ is the absolute value of the difference between intensity *q*_*ij*_ and its local mean value ${\overset{⌢}{q}}_{ij}$. The false component in the neutrosophic domain is calculated as

(5) $$\begin{array}{c} FC=1-TC \end{array}$$

**Figure 1:**
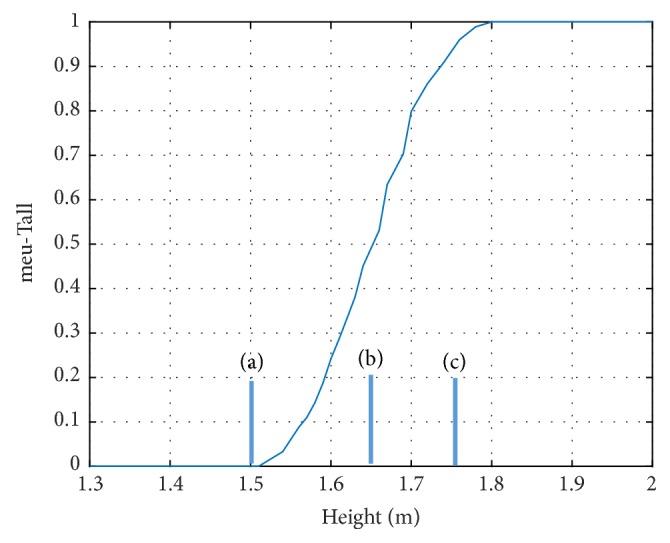
S-function.

*(2) Map Image and Decide *{*T*, *F*}. Consider an image A, P(x, y) is a pixel in the image, and (x, y) is the position of this pixel. A 5x5 mean filter (the size of filter may fluctuate contingent upon the measure of the input image) is applied to A to evacuate noise and make the image uniform. Next, the image is changed by utilizing the S-function:

(6) Tx,ySgxy,a,b,c=00≤gxy≤agxy−a2b−ac−aa≤gxy≤b1−gxy−c2c−bc−ab≤gxy≤c1gxy≥c

where *g*_*xy*_ is the intensity value of pixel P(i, j). Factors a, b, and c are the parameters that evaluate the state of the S-function as appeared in Figure 1.

Estimations of parameters a, b, and c can be computed by utilizing the simulated annealing method [32]. However, the simulated annealing algorithm is quite time-consuming. Thus, we utilize another histogram-based technique to compute a, b, and c [33].

(1) Calculate the histogram of the image.

(2) Find the local maxima of the histogram: His_max_(g_1_), His_max_(g_2_),…His_max_(g_n_).

Calculate the mean of local maxima:

(7) $$\begin{array}{c} \bar{His_{max}\left(g\right)}=\frac{\sum_{i=1}^{n}His_{max}\left(g_{i}\right)}{n} \end{array}$$

(3) Find the peaks greater than $\bar{His_{max}(g)}$; let the first peak be *g*_min_ and the last peak be *g*_max_.

(4) Define low limit B1 and high limit B2:

(8) ∑i=gminB1Hisi=f1∑i=B2gmaxHisi=f1

where the data misfortune is permitted in the range [*g*_max_, *B*_1_] and [*B*_2_, *g*_min_], which is in *f*_1_ (*f*_1_=0.01 in the experiments).

(5) Compute *a* and* c*:

(9) $$\begin{array}{c} a=\left(\;1-f_{2}\;\right)\left(\;g_{1}-g_{min}\;\right)+g_{min} \end{array}$$

         * if *(*a* > *B*_1_)* then* *a* = *B*_1_

(10) $$\begin{array}{c} c=f_{2}\left(\;g_{max}-g_{n}\;\right)+g_{n} \end{array}$$

*If *(*c* > *B*_2_),* then* *c* = *B*_2_, where *f*_2_= 0.01, and *B*_1_ and *B*_2_ are utilized to keep away from imperative data misfortune. The intensity less than *B*_1_ is considered as background, and the intensity more than *B*_2_ is considered as noise.

(6) Compute parameter b by utilizing the most extreme entropy central [34].

(11) $$\begin{array}{c} H\left(\;x\;\right)=\frac{1}{M\times N}\overset{M}{\underset{i=1}{\sum }}\overset{N}{\underset{j=1}{\sum }}S_{n}\left(\;T\left(\;x,y\;\right)\;\right) \end{array}$$

where* Sn* is a Shannon function defined as

(12) SnTx,y=−Tx,y−1−Tx,ylog2⁡1−Tx,yx=1,2,…,M,  y=1,2,…N

The maximum entropy principle expresses that the more noteworthy the entropy is, the more data the framework includes [35]. To determine the optimal b try every* b∈[a +1,c -1]*. The optimal b will produce the largest* H(X)*:

(13) HmaxX,a,bopt,c=max⁡HX;a,b,c ∣ gmin≤a<b<c≤gmax

After* a, b*, and *c* are calculated, the image can be mapped from the intensity domain *g*_*xy*_ to the new domain T (x, y). Use intensification transformation to enhance the image in the new domain [36]:

(14) ETx,y=2T2x,y0≤Tx,y≤0.5ETx,y=1−21−Tx,y20.5<Tx,y≤1

Then image sharpening stage involves applying unsharp mask [36] image sharping technique on the output of image enhancement stage.

#### 2.2.2. Speckle Noise Reducing (SRAD)

SRAD is a type of filters generally utilized for removing speckle noise in ultrasound images and it can save edges, as well as enhances edges. SRAD is a Partial Differential Equation (PDE) that had been adjusted by Yu and Action [7] from anisotropic diffusion filter to fit the speckle noise produced by the ultrasound image. In this algorithm, Instantaneous Coefficient of Variation (ICOV) is utilized to separate the edge regions in the image. ICOV is given by

(15) $$\begin{array}{c} q\left(\;x,y;t\;\right)=\sqrt{\frac{\left(1/2\right){\left(\nabla I/I\right)}^{2}-\left(1/16\right){\left(\nabla^{2}I/I\right)}^{2}}{{\left[1+\left(1/4\right)\left(\nabla^{2}I/I\right)\right]}^{2}}} \end{array}$$

where ∇and∇^2^ denote the gradient and the Laplacian, separately.

(16) ∇gIi,jn=Ii+1,jn−Ii,jnh,Ii,j+1n−Ii,jnh

(17) ∇LIi,jn=Ii,jn−Ii−1,jnh,Ii,jn−Ii,j−1nh

(18) ∇2Ii,jn=Ii+1,jn+Ii−1,jn+Ii,j+1n+Ii,j−1n−4Ii,jnh

ICOV indicate the high value in the edge region and low value in the homogeneous region.

(19) Cq=11+q2x,y;t−qo2t/qo2t1+qo2t

where *q*_*o*_*(t)* is the scale factor of speckle utilized in the dispersion coefficient* C(q).* Equation (19) controls the measure of smoothing in the homogeneous region (17). In this manner, the speckle scale function removes the noise from the homogeneous region.

(20) $$\begin{array}{c} q_{o}\left(\;t\;\right)=\sqrt{\frac{Var\left[Z\left(t\right)\right]}{\bar{Z\left(t\right)}}} \end{array}$$

where* Var[Z(t)]* and $\bar{Z(t)}$ denote the intensity variance and mean over a homogenous region at t.

### 2.3. Seed Point Generation Stage

This stage is started by dividing the thyroid ultrasound nodule images into nonoverlapped square blocks of size 11 by 11 pixels. The Radon transform and HOS were implemented on each block separately to calculate 3^rd⁡^ order entropies at different angles.

#### 2.3.1. Radon Transform

Radon transform is generally utilized in processed tomography to make an image from the dispersing information related to cross-sectional outputs of an object. It transforms the two-dimensional images with lines into a domain of possible line parameters, where each line in the image will give a pinnacle situated at the comparing line parameters [37]. Subsequently, the lines of the images are transformed into the points in the Radon domain. An equation of the line can be expressed as

(21) $$\begin{array}{c} r=x\cos\theta +y\sin\theta \end{array}$$

where* θ* is the small angle and *r* is the small distance to the origin of the coordinate system. Given a function g(x, y), Radon transform is defined as

(22) $$\begin{array}{c} R\left(\;r,\theta \;\right)=\overset{+\alpha }{\underset{-\alpha }{\int }}A\left(\;r\cos\theta -s\sin\theta ,r\sin\theta -s\cos\theta \;\right)ds \end{array}$$

Equation (22) depicts the vital along a line through the image, where *r* is the distance of the line from the origin and *θ* is the angle from the horizontal. So, radon transform changed 2D signal into the 1D parallel beam projections, at different angles *θ*.

#### 2.3.2. Higher Order Spectra Entropy (HOSE)

Higher request spectra are nondirect strategies, characterized to be spectral representations of higher request cumulants (i.e. c_1_, c_2_, and c_3_) of a random process [14]. Both amplitude and phase information for a given signal appear.

The mean value (m) and variance (*σ*_*R*_^2^) are computed by utilizing second-order statistics. They are described by expectation operation as

(23) mr=ER

(24) σR2=ER−mr2

where r is a discrete time signal, and the second-order moment autocorrelation function can be defined as

(25) $$\begin{array}{c} m_{r}^{2}\left(\;i\;\right)=E\left[\;R\left(\;n\;\right).R\left(\;n+i\;\right)\;\right] \end{array}$$

Thus, HOS is composed of moment and cumulant spectra. They are utilized for both deterministic signals and random processes [38]. The third- and fourth-order cumulant spectra are characterized as bispectrum and the trispectrum, respectively [14].

Bispectrum is the Fourier transform of the third order correlation of the data utilized in this work to determine the features is given by

(26) $$\begin{array}{c} B\left(\;f_{1},f_{2}\;\right)=E\left[\;R\left(\;f_{1}\;\right)R\left(\;f_{2}\;\right)R^{*}\left(\;f_{1}+f_{2}\;\right)\;\right] \end{array}$$

where *R*(*f*) is the Fourier transform of the random signal *R*(*nT*),* n* is an integer index,* T* is the sampling interval, and* E*[.] refers to nondeterministic signals (i.e., expectation operation).

Features are utilized in our work based on the integrated bispectrum along the dashed line with slope = r. The frequency (f) normalized by the Nyquist frequency to be between 0 and 1.

**Figure 2:**
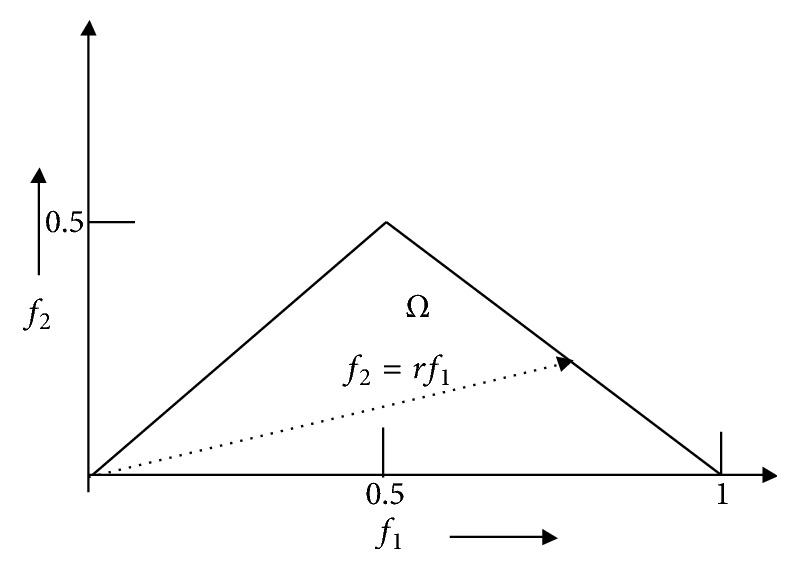
The bispectrum for real signal.

HOS will give information about signal wave shape. If there is no Bispectral associating, the bispectrum of a real signal is uniquely defined with the triangle:

(27) $$\begin{array}{c} 0\leq f_{2}\leq f_{1}\leq f_{1}+f_{2}\leq 1; \end{array}$$

parameters are obtained by integrating along the straight lines passing through the origin in bifrequency space. The district of calculation and the line of integration are depicted in Figure 2.

In this work, we calculated these features within the region Ω.

(1) The Bispectral phase entropy (*P*_*he*_):
  (28) $$\begin{array}{c} P_{he}=\sum p\left(\;w_{n}\;\right)\log p\left(\;w_{n}\;\right) \end{array}$$
(2) Bispectral entropy (*P*_1_):
  (29) $$\begin{array}{c} P_{1}=-\sum_{n}p_{i}\log\left(\;p_{i}\;\right) \end{array}$$
where *p*_*i*_ = |*B*(*f*_1_, *f*_2_)|/∑_*Ω*_|*B*(*f*_1_, *f*_2_)|
Ω is the region as shown in Figure 2.
(3) Bispectral Squared Entropy (*P*_2_):
  (30) $$\begin{array}{c} P_{2}=-\sum_{n}p_{n}\log\left(\;p_{n}\;\right) \end{array}$$
where *p*_*n*_ = |*B*(*f*_1_, *f*_2_)^2^|/∑_*Ω*_|*B*(*f*_1_, *f*_2_)^2^|
(4) Bispectral Cubic Entropy (*P*_3_):
  (31) $$\begin{array}{c} P_{3}=-\sum_{n}p_{q}\log\left(\;p_{q}\;\right) \end{array}$$
where *p*_*q*_ = |*B*(*f*_1_, *f*_2_)^3^|/∑_*Ω*_|*B*(*f*_1_, *f*_2_)^3^|.

In our work, we have extracted the four bispectrum invariants, depicted over every radon-transformed thyroid nodule image.

#### 2.3.3. Proposed System

**Figure 3:**
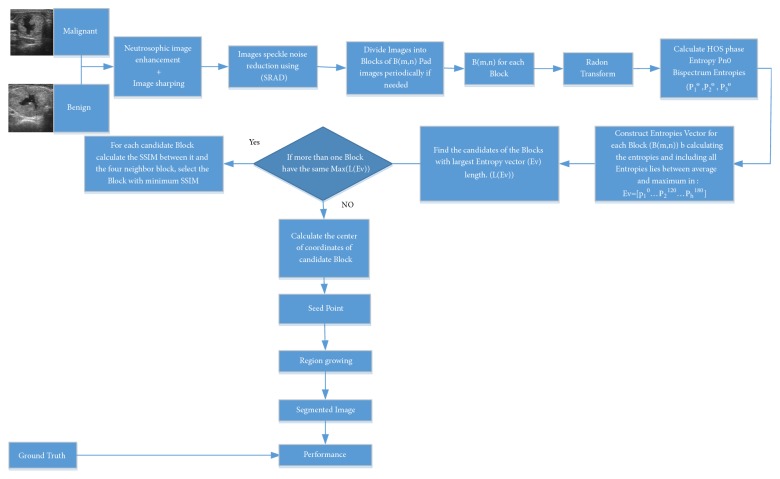
The flowchart of the proposed system.

The block diagram of the proposed system which is depicted in Figure 3. It is started with image preprocessing stage where the entire set of thyroid nodule images (Benign and malignant) is preprocessed by enhancing images visual contrast using neutrosophic based image enhancement followed by image sharpening technique to highlight images edges. Then image speckle noise reduction block using SRAD applied on visually enhanced ultrasound images. The images then are subdivided into equally sized nonoverlapped square blocks B(m,n); then the 3rd order HOSE cumulants entropies at different angles are calculated after applying the Radon transform on the preprocessed ultrasound image blocks. The seed selection process is then started by constructing the entropies vector E_v_ for every image block which includes entropies values from different categories (Bispectral phase entropy (P_he_), Bispectral Entropy (P_1_), Bispectral Squared Entropy (P_2_), and Bispectral Cubic Entropy (P_3_)) at different angles (0°,60°,120°,180°) within the range between maximum and average values calculated from all image blocks. The candidate block then is selected by considering the longest E_v_ vector: Max(L(E_v_)), and hence the center of the candidate block is calculated and considered as a seed point. In the case of more than one candidate block (having E_v_ with the same length),

(32) MaxLEv1MaxLEv2=…..MaxLEvn

The one with highest Structures Similarity Index (SSIM) is becoming the candidate block. The SSIM used for measuring the similarity between two images (or image blocks). The SSIM index is a full reference metric. Thus, if there is a higher amount of homogeneity between the candidate block and its neighbors block its center is selected as a seed. After seed selection, the region growing technique is applied to each thyroid nodule ultrasound images to extract the suspicious area. The ground truth manually segmented images by the specialist are compared with the automatically segmented images and, finally, a group of measurements was carried out to evaluate the output of the proposed system.

### 2.4. Performance Measures

In this work to explore the performance of segmentation methods, both area-based and boundary-based metrics have been utilized. Area based error metrics, True Positive (TP), False Positive (FP), and Dice Coefficient (DC) have been employed. The boundary-based error metrics, for example, Hausdorff Distance (HD), are utilized to decide the possible disagreement over two curves [39].

## 3. Results and Discussion

The proposed segmentation scheme is called seed selection based on higher order spectra (SSHOS). Five real thyroid ultrasound images from dataset are selected and displayed from the used dataset. All images have low contrast with weak boundaries between nodules and abutting tissues.

Table 2 shows the values of maximum SSIM calculated for the 4-nighbour blocks for the candidate blocks. It is clear that the image 28-benign has two candidate blocks (137,187) (see Table 1). The block numbered 187 is selected because it has higher SSIM as compared to the block 137. And this is obvious in Table 2. The Max Neighbor SSIM is calculated by summing out all 4- neighbor's SSIMs.

**Figure 4:**
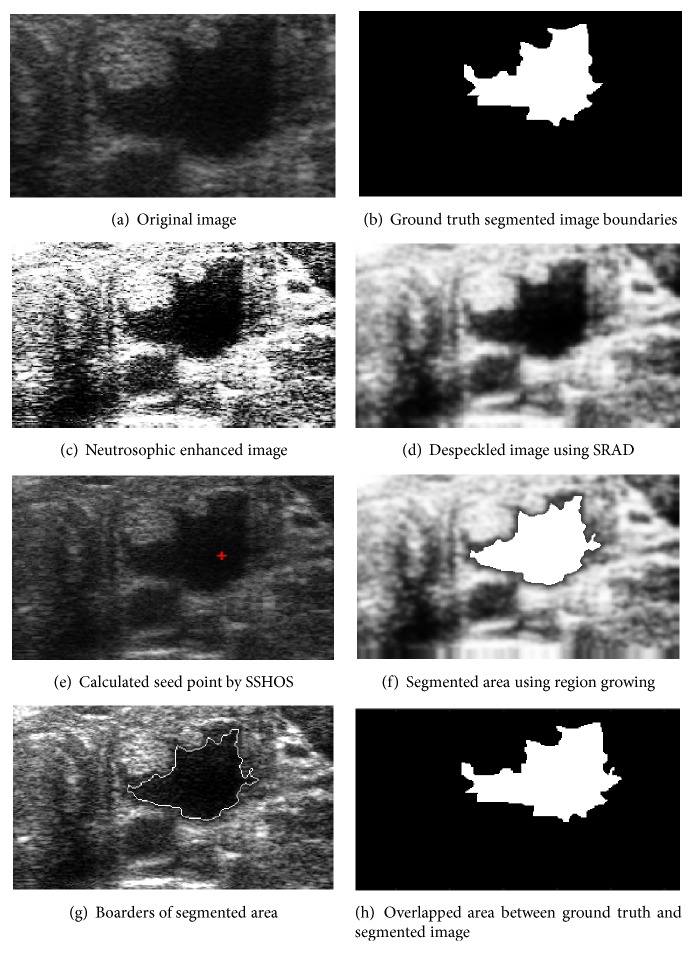
Ultrasound image (image 12 M).

**Figure 5:**
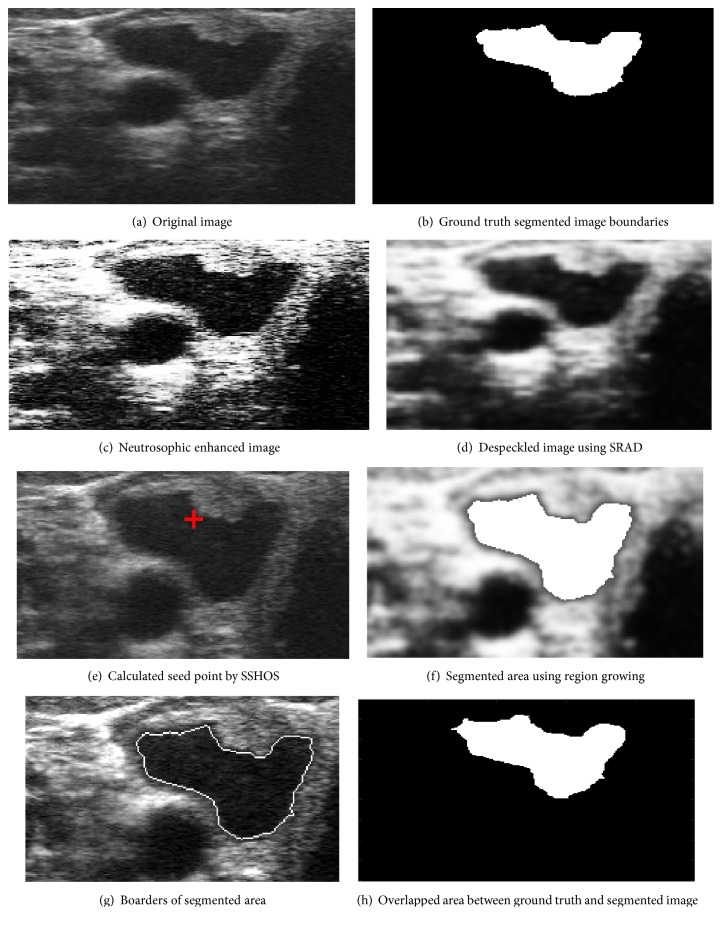
Ultrasound image (image 22B).

**Figure 6:**
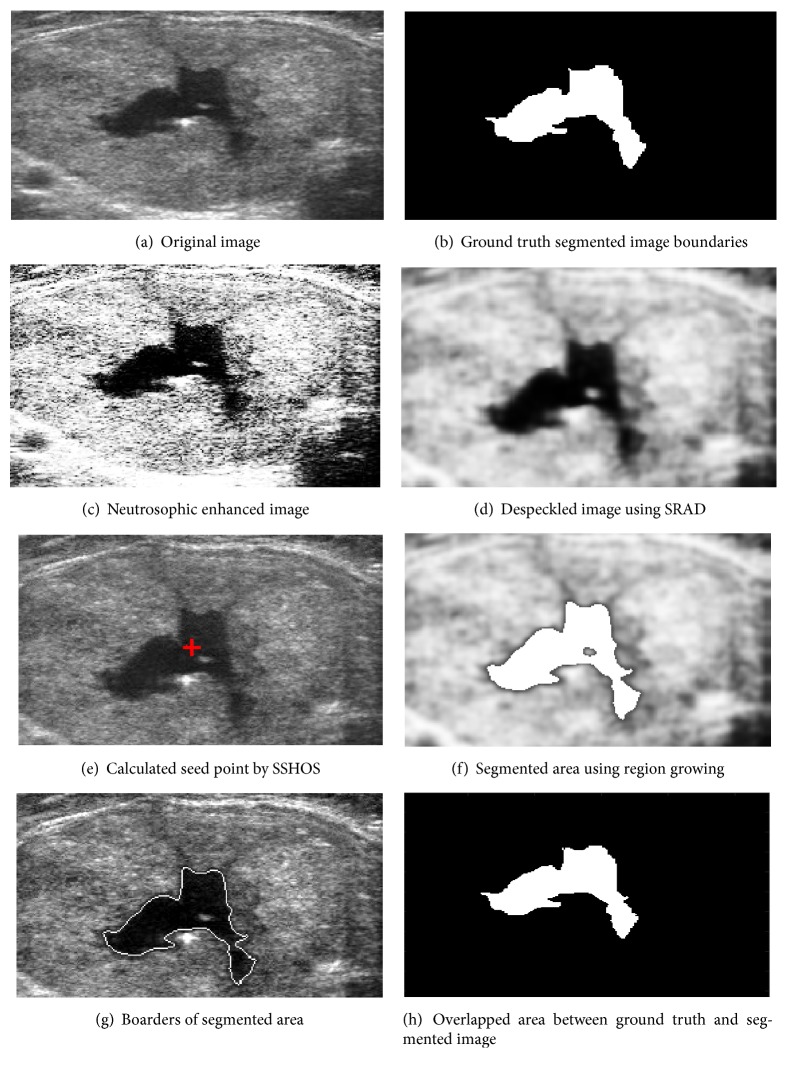
Ultrasound image (image 28B).

**Figure 7:**
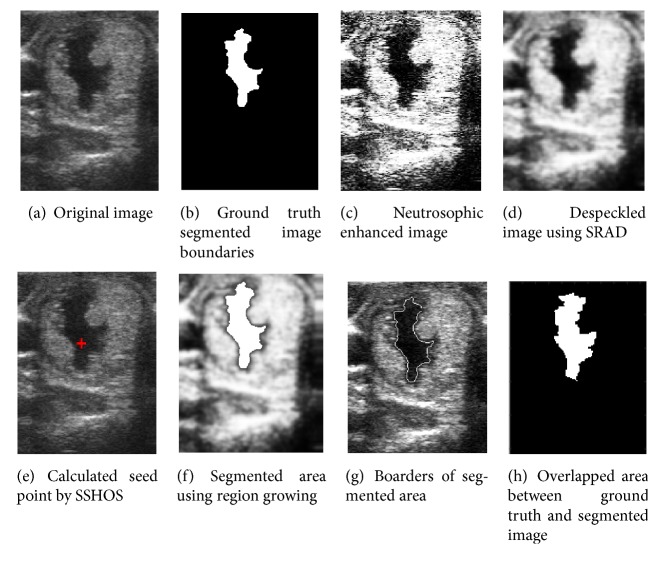
Ultrasound image (image 31M).

**Figure 8:**
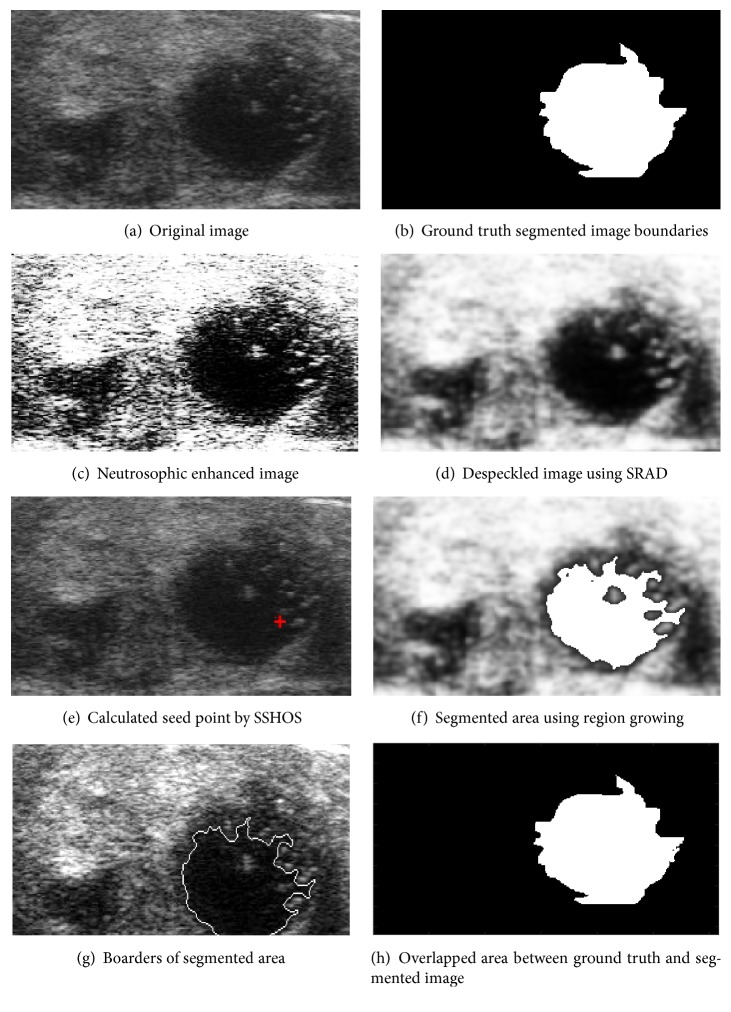
Ultrasound image (image 48B).

Figure 4(a) shows an original ultrasound image.  Figure 4(b) demonstrates ground truth segmented image. Neutrosophic image enhancement and sharpening is introduced in Figure 4(c); Figure 4(d) represented despeckled image utilizing SRAD method. Figures 4(e) and 4(f) demonstrate the visual consequences of SSHOS and segmented sectioned utilizing region growing. It is additionally demonstrating that SSHOS can be utilized for accurate segmentation and separation of particular tissues.  Figure 4(g) illustrated that the SSHOS can better preserved the nodule's boundaries in thyroid ultrasound image while overlapped area between ground truth and segmented image clearly showed up in Figure 4(h). Figures 5–8 show the same details as presented by Figure 4 for the other images listed in Table 1.

**Figure 9:**
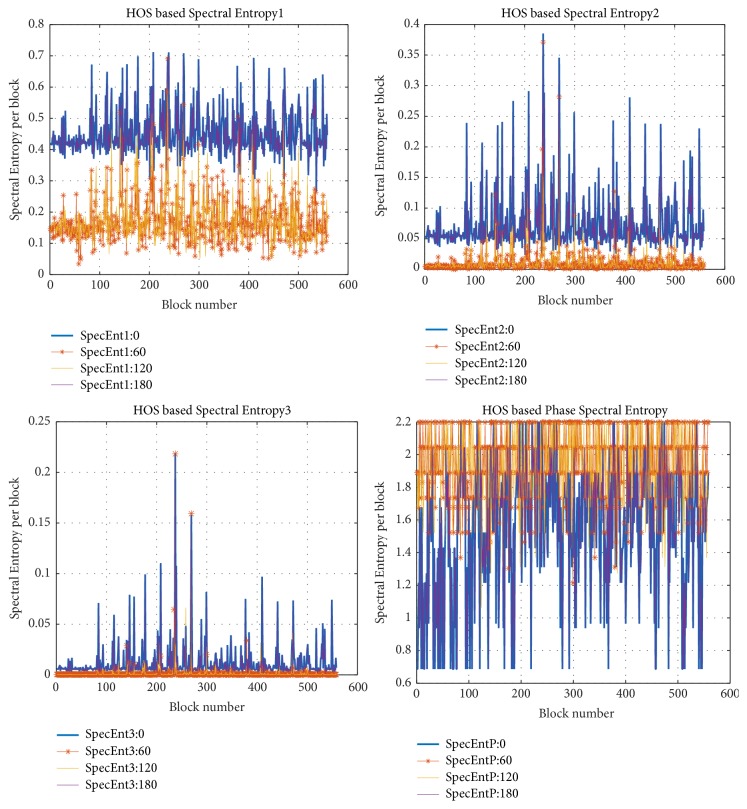
HOSE for different blocks and angles in image (12-malignant).

Figures 9(a), 9(b), 9(c), and 9(d) show graphical illustration for the spectral entropies (P1, P2, P3, and Pph) at different angles for the image (12-malignant). It is clear that the values of different entropies at block numbered (237) are higher than the other blocks entropies. So it is selected as a candidate block automatically.

The performance of segmentation method has been compared with other methods which were applied on the same data set of thyroid ultrasound images as listed in Table 3. As apparent from results, it is seen that SSHOS outperforms all other methods by accomplishing highest qualities regarding TP, SI. The larger estimations of area-based metrics created by SSHOS guarantee more similarity between the regions segmented by segmentation methods. The SSHOS uncovers a change in FP and HD when contrasted with different techniques. Moreover, smallest HD and FP determines the prevalence of proposed techniques as looked at over different methods. The SSHOS is superior to the greater part of different techniques as far as FP, TP, DC, SI, and HD.

The performance of SSHOS with or without speckle reduction is additionally demonstrated by the boundary error measurements as well, which demonstrate that the forms created by SSHOS with or without speckle reduction are significantly nearer to the manual depictions as given in Table 5. As obvious from these outcomes, it is seen that SSHOS can be applied without speckle reduction for the nodule segmentation. All these results justify the great indeterminacy dealing with capacity of neutrosophic domain.

Furthermore, SSHOS can prevent leakage through weak edges resulting in exact extraction of nodule boundaries by dealing with the intensity in homogeneity well. The mean values of TP, FP, DC, SI, and HD acquired with SSHOS are (96.44 ± 3.01%), (3.55 ± 1.45%), (92.24 ± 6.47%), (80.57 ± 1.06%), and (0.42 ± 0.24 pixels). For the most part, SSHOS join to higher qualities than [21, 40].

Table 4 shows a comparison between the proposed method with and without neutrosophic enhancement. It is clear that a degradation in the TP, FP, SI, and all other measures.

Table 5 also shows a comparison between the proposed method (SSHOS) with and without speckle noise reduction. It is clear that the noise reduction technique (SRADA) improves system performance.

## 4. Conclusions

The goal of this study is to improve a robust algorithm for segmenting thyroid nodules on ultrasound image that is a unique challenge in ultrasound segmentation. The result showed that our algorithm is one of the best automatic segmentation methods for thyroid nodules on ultrasound images. In seed selection based on Higher Order Spectra (SSHOS), however, the initial contours are automatically identified very close to the actual thyroid gland nodule boundaries, which can be quickly refined by the level set. Experiments (SSHOS) are highly efficient, robust, and accurate. The experimental results show that SSHOS has better performance results as compared to other methods. It can also prevent border leakage into adjacent tissues and smooth the background.

SSHOS also needs no training and user input. In addition, despite intensity variations, the method can reveal thyroid nodule borders. The higher values of quality meters achieved by the SSHOS method over other state-of-the-art methods are recommended in medical practice.

Thus, without any human intervention, a fully automated CADe system for segmenting nodules in thyroid ultrasound images was developed. It can be used as a second tool for assisting endocrinologists in the automated and accurate delineation of thyroid nodules in ultrasound images. This helps reduce the number of false positives and improves accurate thyroid nodules detection.

Further research may be extended to validate the proposed system for three-dimensional ultrasound images and Doppler ultrasound images, since this work deals only with B-mode ultrasound images. This system still needs to explore other imaging modalities.

## Figures and Tables

**Table 1:** Depicts the five-selected images with their candidates block and blocks center.

Image name	Best Block	No. of Blocks	Block XY-Position	Center point(s)
12-malignant	237	18×31	(8,20)	(83,215)
22-benign	78	19×22	(4,12)	(39,127)
28-benign	(137, 187)	(19×25,19×25)	(6,12), (8,12)	(61,127), (83,127)
31- malignant	180	19×24	(10,9)	(105,94)
48-benign	302	17×28	(11,22)	(116,237)

**Table 2:** The SSIM for the 4-neighbor blocks.

Image name	Max Neighbor SSIM	SSIM BLK1	SSIM BLK2	SSIM BLK3	SSIM BLK4
12-malignant	3.467184				
22-benign	1.64305				
28-benign	(0.9594,2.2008)	(0.0443,0.443)	(0.3386,0.5748)	(0.19089,0.55709)	(0.4742,0.62586)
31-malignant	2.160953				
48-benign	1.879466				

**Table 3:** Performance measures of segmentation method (SSHOS) of dataset.

Scheme Name	HD (Pixels)	TP (%)	FP (%)	SI (%)	Dice coefficient	Density	Area	density k	area k
SSHOS	0.42 ± 0.24	96.44 ± 3.01	3.55 ± 1.45	80.57 ± 1.06	92.24 ± 6.47	0.60 ± 0.17	3123 ± 950	0.60 ± 0.19	3627 ± 1313
Poonguzh et al. [21]	0.66 ± 0.22	93.51 ± 5.94	6.48 ± 3.94	67.85 ± 1.37	80.85 ± 9.04	0.97 ± 0.09	5122 ± 1049	0.80 ± 0.05	6727 ± 1787
Mohammed et al. [40]	0.68 ± 0.28	52.26 ± 4.71	47.73 ± 4.95	51.44 ± 3.01	67.93 ± 2.19	0.76 ± 0.01	4772 ± 699	0.611 ± 0.19	2570 ± 1378
NDRLS [5]	0.2 ± 0.82	95.4 ± 3.5	7.3 ± 5.3		94.2 ± 4.6				

**Table 4:** Performance measures of segmentation method (SSHOS) with and without enhancement images.

Scheme Name	HD (Pixels)	TP (%)	FP (%)	SI (%)	Dice coefficient	density	area	density k	area k
SSHOS	0.42 ± 0.24	96.445 ± 3.01	3.55 ± 1.45	80.57 ± 1.06	92.24 ± 6.47	0.60 ± 0.17	3123 ± 950	0.6086 ± 0.195	3627 ± 1313
without neutrosophic enhancement	0.38 ± 0.12	95.82 ± 3.63	4.17 ± 3.12	72.06 ± 9.55	85.77 ± 6.11	0.69 ± 0.08	2466 ± 1607	0.68 ± 0.17	3175 ± 1765

**Table 5:** Performance measures of segmentation method with and without speckle noise reduction.

Scheme Name	HD (Pixels)	TP (%)	FP (%)	SI (%)	Dice coefficient	Density	area	density k	area k
Without speckle reduction	0.51 ± 0.10	94.19 ± 5.25	5.80 ± 2.56	83.35 ± 1.71	87.92 ± 10.30	0.88 ± 0.10	5923 ± 1850	0.77 ± 0.25	6349 ± 1409
SSHOS	0.42 ± 0.24	96.44 ± 3.01	3.55 ± 1.45	80.57 ± 1.06	92.24 ± 6.47	0.60 ± 0.17	3123 ± 950	0.60 ± 0.19	3627 ± 1313

## Data Availability

We took all data (images) for both Benign and Malignant states in TDID (Thyroid DIgital Image database) which consists of a set of B-mode ultrasound images from the website of Computer Imaging & Medical Applications Laboratory, Universidad Nacional de Colombia Laboratory.
