# Corrigendum to “A Novel Neutrosophic Method for Automatic Seed Point Selection in Thyroid Nodule Images”

**DOI:** 10.1155/2019/4728705

**Published:** 2019-08-04

**Authors:** S. O. Haji, R. Z. Yousif

**Affiliations:** Department of Physics, College of Science, Salahaddin University, Erbil, Iraq

In the article titled “A Novel Neutrosophic Method for Automatic Seed Point Selection in Thyroid Nodule Images” [1] there was an error in the authors' affiliation. The corrected affiliation is shown above.
